# Supplementary material for: Seven-CpG DNA Methylation Age Determined by Single Nucleotide Primer Extension and Illumina’s Infinium MethylationEPIC Array Provide Highly Comparable Results
Source: Front Genet. 2022 Jan 17;12:759357. doi: 10.3389/fgene.2021.759357 (PMC8802213; doi:10.3389/fgene.2021.759357)
Supplement: Supplementary file 1 [file DataSheet1.pdf]

Seven-CpG DNA Methylation Age Determined by Single Nucleotide Primer Extension and Illumina's Infinium MethylationEPIC Array Provide Highly Comparable Results

Valentin Max Vetter[1,2], Christian Humberto Kalies[1], Yasmine Sommerer[3], Lars Bertram[3,4], Ilja Demuth[1, 5]

[1]Charité – Universitätsmedizin Berlin, corporate member of Freie Universität Berlin and Humboldt-Universität zu Berlin, Department of Endocrinology and Metabolic Diseases (including Division of Lipid Metabolism), Biology of Aging working group, Augustenburger Platz 1, 13353 Berlin, Germany

[2]Department of Psychology, Humboldt University Berlin, Berlin, Germany

[3]Lübeck Interdisciplinary Platform for Genome Analytics (LIGA), University of Lübeck, Lübeck, Germany

[4]Center for Lifespan Changes in Brain and Cognition (LCBC), Dept of Psychology, University of Oslo, Oslo, Norway

[5]Charité - Universitätsmedizin Berlin, BCRT - Berlin Institute of Health Center for Regenerative Therapies, Berlin, Germany

Corresponding author:
Ilja Demuth (Ph.D.)
Charité - Universitätsmedizin Berlin
Lipid Clinic at the Interdisciplinary Metabolism Center,
Biology of Aging Group
Augustenburger Platz 1
13353 Berlin
Email: ilja.demuth@charite.de
Phone: ++49 30 450 569 143
FAX: ++49 30 450 566 904

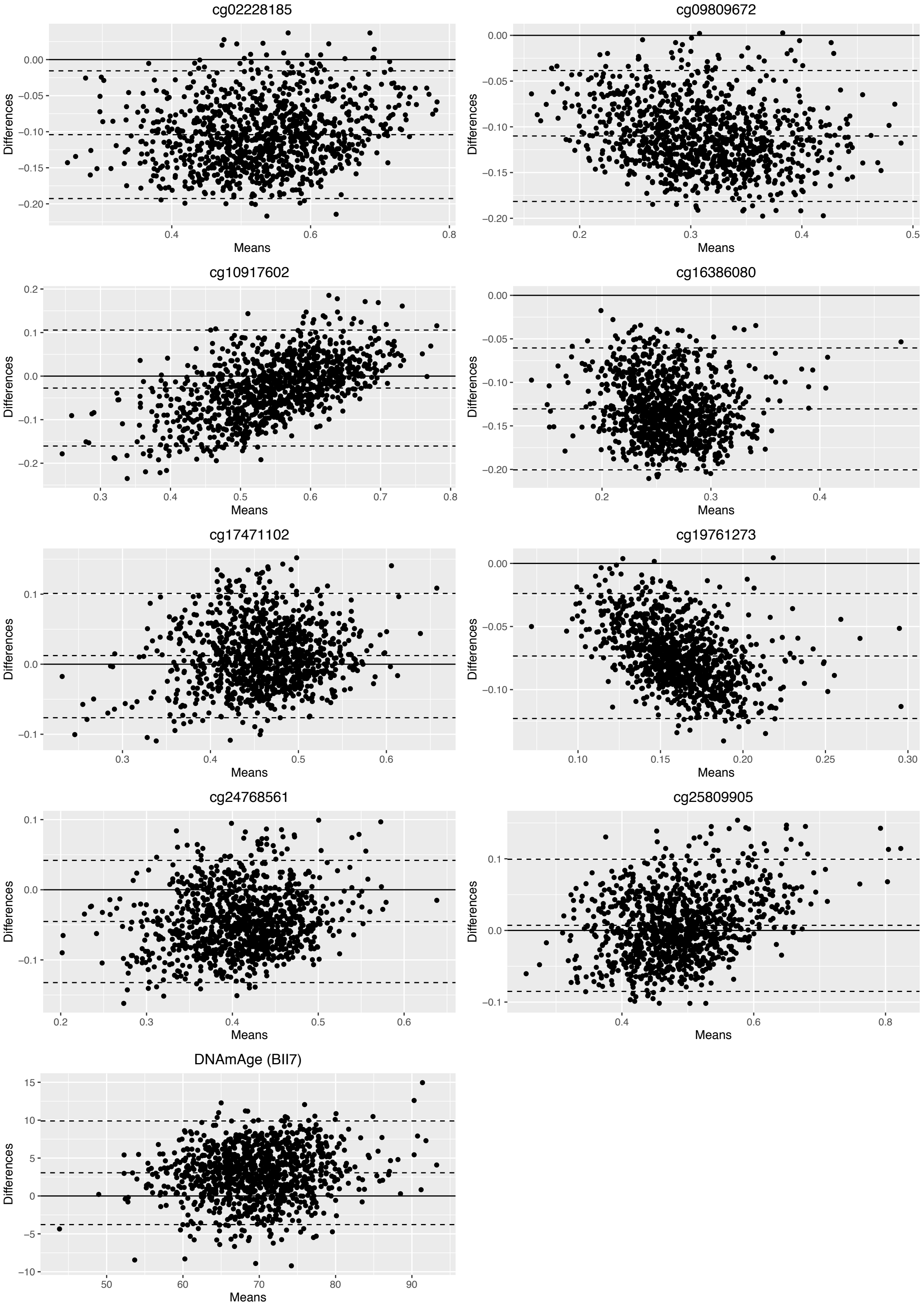

Supplementary Figure S1: Bland-Altman plots of the eight individual CpG sites and the resulting 7-CpG clock DNAm age. Cg10917602 was measured but is not included in the calculation of the 7-CpG clock.

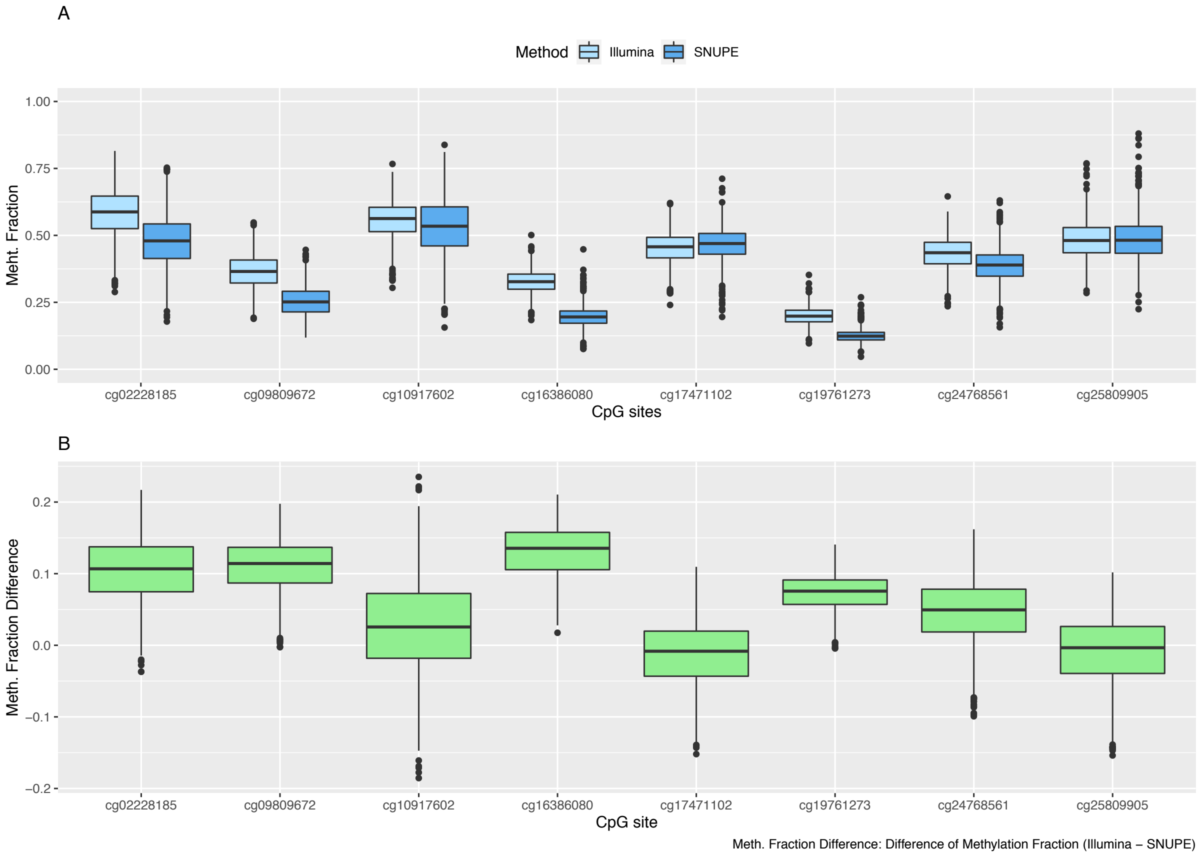

Supplementary Figure S2: Boxplots of methylation fraction measured by the SNuPE and EPIC array method (A) and difference between both methods (B). Cg10917602 was measured but is not included in the calculation of the 7-CpG clock. In contrast to Figure 1 of the main manuscript it is included here as well.

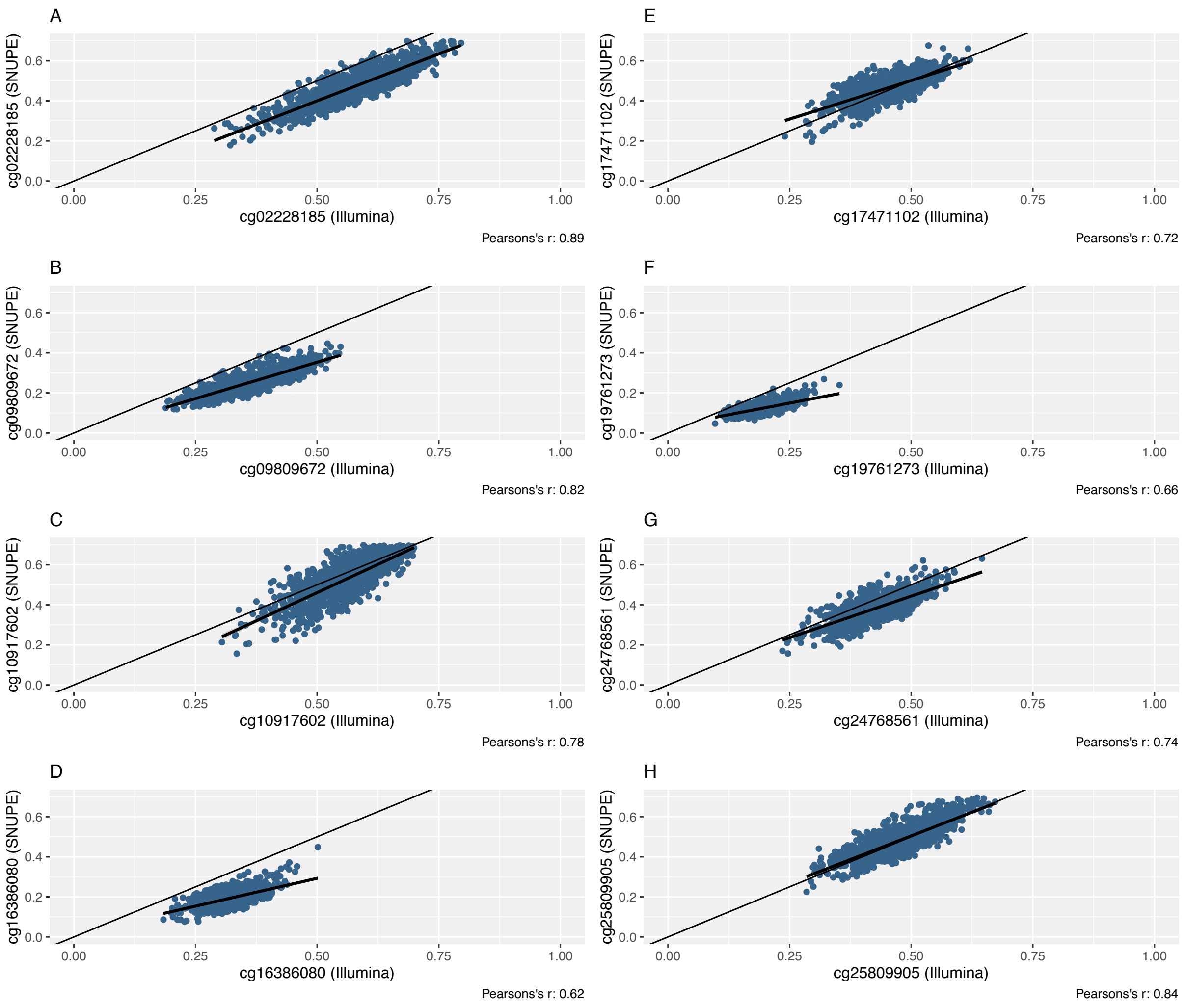

Supplementary Figure S3: Scatterplots of the methylation fraction of eight CpG sites that were measured with the SNuPE and EPIC array (Illumina) method (A-H). The line of equality (thin) and the regression line (bold) are displayed. In contrast to Figure 2 of the main manuscript, cg10917602 is included here as well.
